# Importance of Regeneration of Head of Femur after Resections of Hip-joint

**Published:** 1918-12

**Authors:** 


**On the Importance of the Regeneration of the Head of the Femur after Wide Resections of the Hip-joint.** By R. LERICHE. *Bulletin de la Société de Chirurgie de Paris*, May 28, 1918.

It is admitted that the head and neck of the thigh-bone never seem to regenerate after having been resected. Dr. LERICHE asserts never having seen even the slightest reappearance of the head of the femur on X-ray photographs. When the head of the femur has been removed, it appears that the section of it and the acetabulum simply unite by strong fibrous ligaments.

Nevertheless, Dr. Leriche considers that it is easy to obtain a very satisfactory regeneration of the head of the femur, even after complete and primary resections, although the contrary is widely admitted. This may be obtained simply by changing the operatory method.

The operation must be carried out in this way: when the joint appears freely, instead of opening the capsule, the neck of the femur must be rugined *from the outside*, beginning by its pretrochanteric insertions, and all around if possible; *the capsule must be pushed back uninjured into the acetabulum.* When this is done as completely as possible, the capsule is incised, the head of the bone disjointed, and the operation continued according to the necessities of each case.

By proceeding in that way, a remarkable regeneration of the head of the femur has been obtained in the case of a man operated upon 5 hours after being wounded. After 35 days, the trochanter was regenerated. After 75 days, the new trochanter was solidly joined to the diaphysis, and the beginning of the head could be detected. After 105 days, the head was quite visible, just in front of the acetabulum. After 164 days, it was 5 or 6 cm. long and penetrated into the acetabulum. This head and the diaphysis are about at a right angle, at the basis of the trochanter.

After 6 months this man can easily walk two miles a day without an apparatus. The shortening of the leg is about 4 cm.

The author thinks this result is due to the fact that he attacks the bone with the rugine, leaving a thin portion of it attached to the periosteum. The cells of that thin portion develop, and are able to regenerate new bony substance.

